# Pathways to Well-Being in Later Life: Socioeconomic and Health Determinants Across the Life Course of Australian Baby Boomers

**DOI:** 10.1007/s12062-015-9132-0

**Published:** 2015-08-19

**Authors:** Hal Kendig, Vanessa Loh, Kate O’Loughlin, Julie Byles, James Y. Nazroo

**Affiliations:** Centre for Research on Ageing, Health, and Well-being, Research School of Population Health, Australian National University, Building 62A, Eggleston Road, Canberra, ACT 0200 Australia; ARC Centre of Excellence in Population Ageing Research (CEPAR), Sydney, Australia; School of Psychology, Brennan MacCallum Building (A18), University of Sydney, Sydney, NSW 2006 Australia; Ageing, Work and Health Research Unit, Faculty of Health Sciences, University of Sydney, 75 East St, Lidcombe, Sydney, NSW 1825 Australia; Research Centre for Gender, Health and Ageing, University of Newcastle, University Drive, Callaghan, NSW 2308 Australia; School of Social Sciences, University of Manchester, Manchester, M13 9PL England UK

**Keywords:** Social determinants, Socioeconomic status, Childhood exposures, Quality of life, Life satisfaction, Social mobility

## Abstract

In many countries like Australia and the United States, baby boomers are referred to as the ‘lucky cohort’, yet there has been little research on the origins and extent of inequalities within this cohort. This study uses path analysis to investigate direct and indirect effects of childhood and adult socioeconomic status and health on two subjective well-being measures: quality of life and life satisfaction. Retrospective life course data were obtained for 1,261 people aged 60 to 64 in the 2011–12 Life Histories and Health survey, a sub-study of the Australian 45 and Up Study. Supporting an accumulation model, the number of negative childhood and adult exposures were inversely related to both types of well-being. Consistent with a critical period model, childhood exposures had small but significant effects on subjective well-being and were relatively more important for quality of life than for life satisfaction. However, these childhood effects were largely indirect and significantly mediated by more proximal adult exposures, providing support for a pathway model. A key implication of this research is that the critical period for later life well-being is significant in adulthood rather than childhood, suggesting that there may be key opportunities for improving individuals’ later life well-being far beyond the early, formative years. This research highlights the importance of understanding how earlier life exposures impact experiences in later life, and investing in health and socioeconomic opportunities to reduce inequalities across all stages of life.

**Electronic supplementary material:**

The online version of this article (doi:10.1007/s12062-015-9132-0) contains supplementary material, which is available to authorized users.

## Introduction

Promoting health and well-being across the life course are policy priorities for governments in countries experiencing structural ageing and ongoing social change. In setting goals for individual and societal ageing, there is increasing recognition of the value of multi-dimensional indicators of well-being including subjective measures that complement more traditional, objective measures of physical health and material wealth (Dolan et al. 2011; Huppert and So 2013; Stiglitz et al. 2009). Social theorists recognize the systematic inequalities of opportunity and disadvantage that accumulate as people move through variable social structures over the life course; further variation is introduced for successive cohorts living lives in different periods of history (Dannefer and Settersten 2010).

Current indicators suggest that the life histories and health outlooks of the baby boom cohorts (born 1946–1965) now entering later life are likely to be very different from earlier cohorts who were born during the Great Depression and World War II (WWII). While the timing and social exposures of the boomer cohorts have varied among countries, their sheer size relative to other cohorts makes this generation particularly influential (Leach et al. 2013). In many countries like Australia, they have been at the vanguard of much social change, experienced a number of social, political, cultural, and economic milestones, and are reaching the ages at which they are most likely to be experiencing a number of important work and health changes as they transition from middle to later life and pass traditional retirement ages (Kendig et al. 2013; O’Loughlin et al. 2010). These boomer cohorts have had full exposure to the post-WWII era inclusive of the education and fertility revolutions of the 1960s, and their experiences will likely shape the future of ageing populations.

In Australia and the United States (US), there is a widespread view that the early baby boom cohort has been advantaged relative to later cohorts (Hamilton and Hamilton 2006; Phillipson et al. 2008), but there has been little attention to the origins of inequalities arising from earlier life experiences within this cohort. This paper begins to address this gap by examining the ways in which subjective well-being on entry to later life is influenced by earlier childhood and adult socioeconomic and health circumstances. The findings are specific to those in the early postwar cohort (born 1947–1951) and their experiences of social and economic change to 2011 when they were aged 60 to 64 years.

### Life Course Approaches

A growing number of European studies provide support for the view that earlier life experiences impact later life outcomes such as health and mortality (Blane et al. 2004; Marmot et al. 2012; Niedzwiedz et al. 2012). Evidence suggests that childhood health and socioeconomic status (SES) have long-term effects that extend into adulthood (Brandt et al. 2012). Drawing on the social determinants of health perspective, a US study found that socioeconomic disparities in childhood are related to childhood health and education which, in turn, relate to occupational attainment and adult health, highlighting the interrelatedness of health and SES across the life course (Haas 2006). These findings are consistent with contemporary life course approaches that argue a person’s developmental trajectory is influenced by both proximal and early life circumstances and resources (Pavalko and Caputo 2013). Life course approaches allow us to recognize the complex interplay between individual life exposures and changing social structures and provide a broad framework for examining the impact of both individual and social factors on health and well-being in later life.

Three key ‘hypotheses’ or causal models often appear in life course research (Hallqvist et al. 2004; Mishra et al. 2009; Niedzwiedz et al. 2012). The most prominent is the accumulation model, which hypothesizes that exposures to advantageous and disadvantageous circumstances throughout the life course have a cumulative, systematic effect on later outcomes irrespective of timing (Dannefer 2003; Mishra et al. 2009). Evidence for the accumulation model has mostly come from research linking SES exposures to physical health outcomes such as self-rated health and functional limitations, suggesting that the greater the number of negative SES exposures, the poorer the outcomes (Grollman 2014; Otero-Rodríguez et al. 2011). Although there have been some mixed research findings suggesting that the risk of poor mental health increases linearly with increasing number of negative SES exposures, particularly amongst men (Singh-Manoux et al. 2004), it remains to be seen whether health and SES exposures also cumulatively impact well-being. Consistent with the accumulation model, the number of disadvantageous or negative SES and health exposures experienced in both childhood and adulthood are expected to be associated with poorer well-being outcomes in later life.

In contrast, critical period (or latent effects) models hypothesize that exposures during a critical life period, most commonly early in life, have a lasting effect on later outcomes independent of exposures outside this critical time (Ben-Shlomo and Kuh 2002). Whilst research generally suggests the greater importance of early SES and health exposures for lifelong health, some early research with pre-boomer cohorts suggests the greater impact of more recent, proximal SES exposures on subjective well-being (Blane et al. 2004; Houle 2011). Similarly, more recent indicators of adult health might also be expected to show stronger relationships with later life well-being than more distal measures of childhood health.

Finally, in pathway and social mobility models, the direction or shape of trajectories across the life course are important such that adult SES and life circumstances may partially or fully mediate the effects of earlier exposures (Laaksonen et al. 2007; Power and Hertzman 1997). Evidence supporting these models includes research linking lower childhood and adult SES to poorer mental health outcomes in middle life (Singh-Manoux et al. 2004), and the strong body of evidence linking childhood health to adult health (Haas 2006; Haas et al. 2012), and health to well-being (Peiró 2006). These effects suggest both a socioeconomic pathway and a health pathway by which the impacts of childhood SES and health would be mediated by adult SES and health, respectively.

Although the three models are sometimes used to set up competing hypotheses, there is recent agreement that these models are not necessarily incompatible, and the often mixed empirical support is likely to be due to the different methods and measures employed (Hallqvist et al. 2004; Niedzwiedz et al. 2012; Singh-Manoux et al. 2004).

### Research on Subjective Well-Being and Life Course Effects

A recent systematic review by Niedzwiedz et al. (2012) reported an overall relationship between SES over the life course and well-being indices including quality of life and life satisfaction, but found mixed support for each life course model. They recommended that future research should try to test all models within a single study and use multiple measures of well-being.

Subjective well-being is a complex, multifaceted construct with measures generally falling within or across two broad categories (Steptoe et al. 2012; Vanhoutte 2014; Vanhoutte and Nazroo 2014). The first is hedonic well-being (Diener et al. 2010; Kahneman and Deaton 2010), which includes emotional (or affective) well-being and evaluative (or cognitive) well-being (also called life evaluation). Emotional well-being refers to the experience of positive or negative emotions and feelings, whereas evaluative well-being refers to more global thoughts about one’s satisfaction with life. The second is eudemonic (or psychological) well-being, involving existential evaluations of self-realization and reflecting the extent that one feels they are living in accordance with their ‘true self’, their personal values, and fulfilling their potential (Ryan and Deci 2001; Ryff et al. 2003).

Despite research linking later life well-being to adult SES (Blane et al. 2007) and health (Jivraj et al. 2014), there is little research on the life course determinants of well-being (Blane et al. 2004), and the effects of childhood SES and health on different types of later life well-being remain unclear. A few recent studies analyzing retrospective life history data collected in the third wave (2008 to 2009) of the Survey of Health, Ageing and Retirement in Europe (SHARE) provide early evidence of the significant effects of childhood SES on satisfaction (Deindl 2013; Niedzwiedz et al. 2014) and quality of life (Wahrendorf and Blane 2014) for individuals aged 50 years and over. In addition, Deindl (2013) found support for a pathway model in which childhood SES had an indirect effect on life satisfaction via late life income, and while childhood conditions remained significant, current income and health had stronger effects on current life satisfaction. Wahrendorf and Blane’s (2014) analysis of SHARE participants born between 1928 and 1947 who were no longer working but had reported working for at least 5 years, provides evidence for the cumulative effects of childhood SES, and the partially mediating role of work histories, on quality of life in older adults. Their findings also suggested that future research should examine educational qualification and functional limitations as additional pathways linking childhood factors to quality of life in older age. This study contributes to this emerging research by examining the relative and cumulative impacts of childhood and adult SES and health on both life satisfaction and quality of life measures within a single study.

### Current Study and the Australian Context

Much of the reviewed research on the social determinants of health and early life course effects has been conducted in longer-established Western countries, such as the US and the United Kingdom (UK), rather than in more recently-developed Western countries, such as Australia and Canada. The latter countries, having had more recent economic growth and different political cultures with fewer class barriers, may be more conducive to social mobility, compared to countries like the US, which have experienced relatively earlier industrial stagnation, limitations on available space and infrastructure growth, privatization and increasing costs of services previously provided by governments. Indeed, prior research has found support for greater intergenerational mobility in countries like Australia and Canada, and less mobility in the US and the UK, at least in terms of sons’ adult earnings compared to that of their father (Hacker and Pierson 2010; Leigh 2007; OECD 2010). This may be in part due to the smaller socioeconomic gaps found in Australia and Canada compared to the US and UK (Bradbury et al. 2011). Further, the retirement plans of older workers in Australia are relatively less influenced by concerns for the costs of healthcare than those in the United States (Sargent-Cox et al. 2012), and Australian baby boomers have been relatively less affected by the 2008 financial crisis and its aftermath compared to their European and North American counterparts (Kendig et al. 2013). These cross-national differences make it particularly important to examine whether the strong life course effects found in prior research also applies to the Australian baby boom cohorts. Consistent with past research (Deindl 2013; Wahrendorf and Blane 2014) and in the context of this ‘lucky’ baby boom cohort experiencing greater social mobility, adulthood may be more critical for later life subjective well-being than one’s early childhood years.

Drawing on the extant literature, this study tests four hypotheses that potentially can extend our understanding of life course influences on well-being in later life. The first two hypotheses test the accumulation and critical period models for subjective well-being, while the last two test the mediation effects predicted by a pathway model. Emerging research on the social determinants of health suggests that childhood SES impacts adult health (Conroy et al. 2010; Luo and Waite 2005), which in turn has been linked to well-being (Mckenzie et al. 2010), and that childhood health impacts adult SES (Haas 2006). However, less is known about whether childhood health directly impacts adult SES independently of childhood SES. Thus, the last two hypotheses also test whether adult SES mediates the effects of childhood health, and whether adult health mediates the effects of childhood SES.

1. An increasing number of negative SES and health factors will be related to poorer well-being.
2. Adult SES and health will be more strongly related to well-being than childhood SES and health.
3. Adult SES will mediate the effects of childhood SES and health on well-being.
4. Adult health will mediate the effects of childhood health and SES on well-being.

## Data and Methods

### Participants and Procedure

The data were collected in the 2011–12 Life Histories and Health (LHH) Survey (Kendig et al. 2014b), conducted as a sub-study of the Sax Institute’s Australian population-based “45 and Up Study” (https://www.saxinstitute.org.au). As the largest cohort study in the Southern Hemisphere with some 267,000 participants, the 45 and Up Study began documenting and tracking the health of residents in the state of New South Wales aged 45 years and over in 2006 (45 and Up Study Collaborators 2008). LHH Survey invitation packs containing participant information, a consent form, questionnaire, life grid and post-paid reply envelope were posted to a random sample of 2,800 individuals from the 45 and Up Study, aged 60–64 years (born 1947–1951) in 2011. Of the 1,507 who returned completed consent forms and questionnaires (54 % response rate), 1,261 participants (84 %) completed the follow-up telephone interview collecting comprehensive work and life history data. Participant characteristics for this sample are provided in Kendig et al. (2014b, Table 2). This analysis uses data from the baseline, self-completed 45 and Up Study questionnaire and the LHH Survey. The 45 and Up Study was approved by the University of New South Wales Human Research Ethics Committee and the LHH Survey was approved by the Human Research Ethics Committees of the University of Sydney and the University of Newcastle.

### Measures

LHH Survey measures were largely based on those in the English Longitudinal Study on Ageing (ELSA; Steptoe et al. 2013) and the baseline 45 and Up Study survey (https://www.saxinstitute.org.au/our-work/45-up-study/). Variables from both surveys have been used extensively in published research (e.g., Jivraj et al. 2014; Majeed et al. 2015), with detailed information provided in the references given above.

#### Subjective Well-Being

Eudemonic well-being was measured with CASP-19, a 19-item scale designed specifically for older adults, assessing four primary domains of individual needs that reflect quality of life: control, autonomy, self-realization and pleasure (Hyde et al. 2003). Participant responses (often, sometimes, not often, never) to each item were summed to form a total score ranging from 0 to 57, with higher scores indicating better well-being. Coefficient alpha was 0.89.

Hedonic evaluative well-being was measured using the 5-item Satisfaction With Life Scale (SWLS; Diener et al. 1985), a widely used measure of subjective well-being assessing overall life satisfaction (Pavot and Diener 2008). A 7-point Likert scale with responses ranging from strongly agree to strongly disagree was used. Total summed scores ranged from 5 to 35, with higher scores indicating greater life satisfaction. Coefficient alpha was 0.90.

#### Childhood SES

The three childhood SES indicators were parental SES, access to books at home at age 10, and school attendance at age 16 (0 = No, 1 = Yes). Parental SES was based on the highest occupation score of either parent using the Australian Socioeconomic Index 2006 (AUSEI06; McMillan et al. 2009), a continuous measure providing scores ranging from 0 (labourers) to 100 (medical practitioners), with higher scores reflecting higher occupation-based SES. The AUSEI06 was derived using 2006 Australian Census data for males and females aged 21-64 in the workforce, including their occupation, education, income, and hours worked. As with other international socioeconomic indices, it is moderately correlated with education and income levels (see McMillan et al. 2009 for further details). Access to books at home is a childhood SES indicator from both ELSA and SHARE life history surveys, which reflects the educational level, cultural capital, and scholarly culture of adults in the household (Cavapozzi et al. 2011; Evans et al. 2010). A single item asked ‘About how many books were there in your home?’ with five response options: 1 = None or very few, 2 = Enough to fill one shelf, 3 = Enough to fill one bookcase, 4 = Enough to fill two bookcases, 5 = Enough to fill three or more bookcases.

To test Hypotheses 1 and 2, respondents received a score from 0 to 3 with higher scores indicating more negative childhood SES exposures: parental SES scores in the bottom tertile, less than one bookcase of books at age 10, and not being at school at age 16.

#### Adult SES

Adult SES was indicated by the respondent’s highest educational qualification (0 = no post-school qualification, 1 = certificate or diploma, 2 = bachelor’s degree or higher), the socioeconomic index (AUSEI06) of their most significant job, and their gross household income in Australian dollars before tax (from 1 = Less than $5,000 per year to 8 = More than $70,000 per year). Given the older age range and varied work histories of participants, the most significant job rather than their last or current job was used as an indicator of adult SES. Participants were asked to think back over all the jobs they had told the interviewer about and to choose the job that made the most significant contribution to their financial resources and helped them most in getting ahead financially.

For Hypothesis 1, adult negative exposures were counted whenever participants scored in the lowest tertile such that having no post-school qualification, an AUSEI06 score for their most significant job and a household income in the lowest tertile (less than $40,000 per year) resulted in a score of 3.

#### Health

Childhood health was indicated with a single item asking ‘Would you say that your health during your childhood was excellent, very good, good, fair, or poor?’ Similarly, adult health was assessed with an item asking ‘Would you say your health is excellent, very good, good, fair, or poor?’ Responses to both items were coded from 1 (excellent) to 5 (poor). Using repeated measures data from the US Panel Study of Income Dynamics (PSID) and unique data from the Health and Retirement Study (HRS), Haas (2006, 2007) found support for the reliability and validity of these broad retrospective reports of childhood health. For Hypotheses 1 and 2, ratings of fair or poor childhood or current health were each coded as negative health exposures.

### Control Variables and Analysis

Missing data were imputed using the IBM SPSS Statistics Version 20 routine for expectation-maximization (EM) estimation, which is recommended over listwise and pairwise deletion (Schafer and Graham 2002). Study variables had 3.7 % or fewer missing values, with the exception of income (16.9 % missing), which often has higher missing values. Those with missing data were more likely to be female, have lower educational qualifications and AUSEI06 scores, and poorer childhood health than those with complete data. There were no substantive differences in the results using the non-imputed data.

**Fig. 1:**
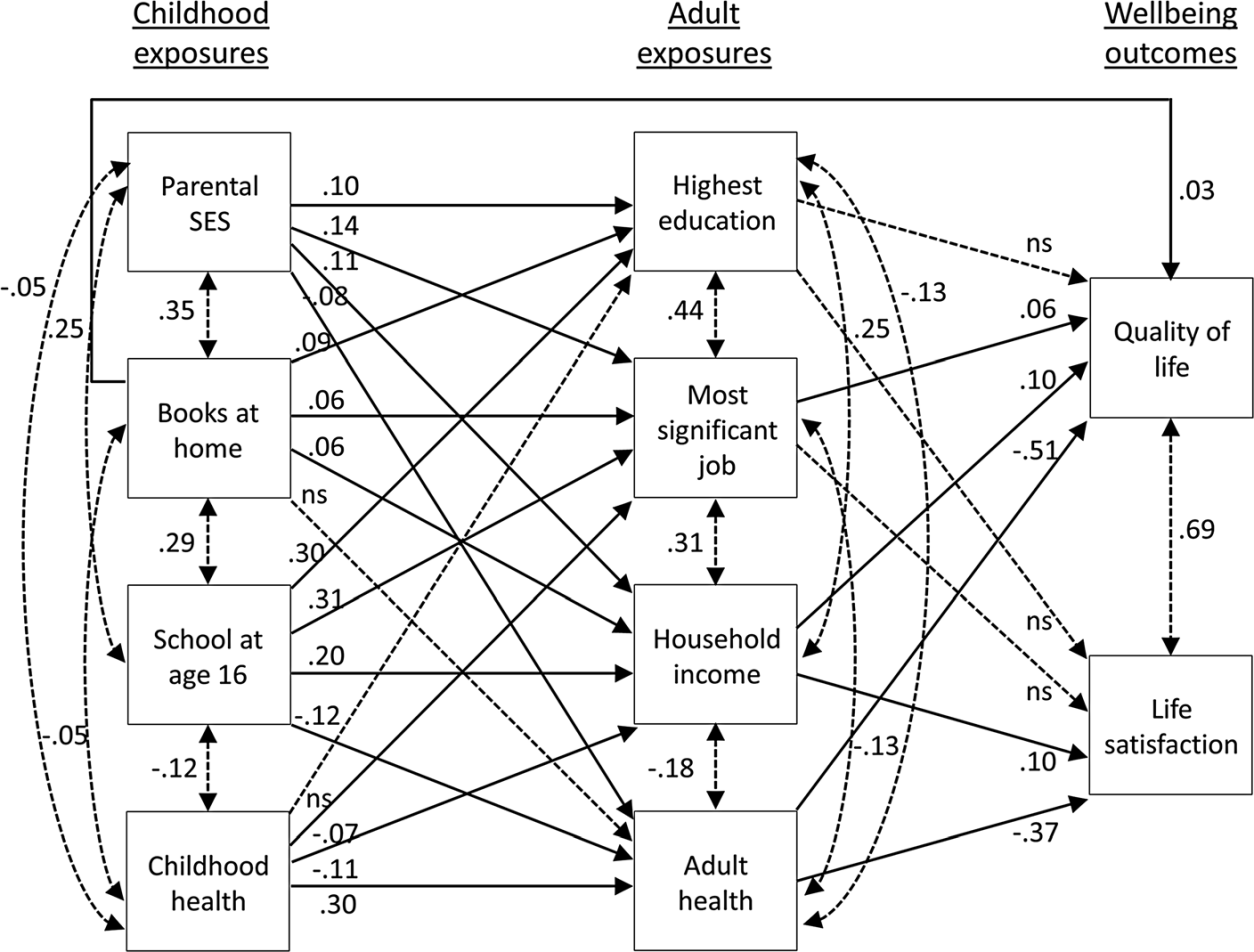
Standardized estimates for full mediation model 2c controlling for age, gender, marital and work status. *Dotted lines with double-headed arrows* indicate correlated factors. *Dotted lines with single-headed arrows* indicate non-significant (ns) paths, *p* > .05

Hypotheses 1 and 2 were tested with correlations and independent-samples *t*-tests, while Hypotheses 3 and 4 were tested using path analysis in Mplus Version 7. A separate path analysis was first run for each dependent variable to test the hypothesized path models, which were based on prior research and theory. Given that the results did not change substantively when both well-being measures were included in the same model, the combined path analysis is reported both for parsimony and to model the significant relationship between the two well-being measures. Though not shown in Fig. 1, the path models controlled for demographic variables associated with well-being (Jivraj et al. 2014; Vanhoutte and Nazroo 2014), including age, gender (0 = male, 1 = female), marital status (0 = not married or de facto, 1 = married or de facto), and work status (0 = not in paid work, 1 = in paid work). Model fit was assessed by comparing the indices recommended by Kline (2005), including the model chi-square (*χ*^2^), root mean square error of approximation (RMSEA <0.08 indicates good fit), comparative fit index (CFI > 0.9 indicates good fit), standardized root mean square residual (SRMR <0.1 indicates good fit), and the Akaike information criterion (AIC). Lower *χ*^2^ (with *p*-values >0.05) and AIC values generally indicate better fit.

## Results

### Number and Timing of Exposures

Over a third (36.3 %) of participants reported no negative childhood SES exposures, 30.4 % reported one, 24.3 % reported two and 9.0 % reported three exposures. Almost half (49.0 %) reported no negative adult SES exposures, 29.0 % reported one, 16.8 % reported two, and 5.2 % reported three exposures. Supporting Hypothesis 1, an increasing number of negative childhood and adult SES exposures showed small but significant associations with poorer quality of life (childhood: *r* = −.14, *p* < .001; adult: *r* = −.29, *p* < .001) and life satisfaction (childhood: *r* = −.10, *p* < .001; adult: *r* = −.22, *p* < .001). The slightly larger correlations of adult exposures with both well-being measures support Hypothesis 2.

Only 7.1 and 12.8 % of the sample reported poor to fair health in childhood and adulthood, respectively. Independent-samples *t*-tests showed that those with poorer childhood health had lower quality of life (*M* = 40.90, *SD* = 9.83), *t* = 4.44, *p* < .001, *d* = .43, and life satisfaction (*M* = 25.34, *SD* = 6.76), *t* = 4.44, *p* = .002, *d* = .31, than those with good childhood health (quality of life: *M* = 44.78, *SD* = 7.84; life satisfaction: *M* = 27.30, *SD* = 5.76). Those with poorer adult health also reported lower quality of life (*M* = 34.51, *SD* = 9.80), *t* = 4.44, *p* < .001, *d* = 1.37, and life satisfaction (*M* = 21.95, *SD* = 7.55), *t* = 4.44, *p* = .002, *d* = .93, than those with good health (quality of life: *M* = 45.97, *SD* = 6.60; life satisfaction: *M* = 27.93, *SD* = 5.14). Following Cohen’s (1988) guidelines, the mean effect sizes (*d*) for adult health may be considered large while those for child health are considered small, supporting Hypothesis 2. Including poor health in the count of childhood and adult exposures yielded similar, but slightly stronger correlations with both quality of life (childhood: *r* = −.17, *p* < .001; adult: *r* = −.40, *p* < .001) and life satisfaction (childhood: *r* = −.11, *p* < .001; adult: *r* = −.30, *p* < .001), suggesting that both health and SES exposures impact later life well-being.

### Testing a Mediated Pathway Model

The descriptive statistics and intercorrelations for the variables used in the path analysis are shown in Table 1. Age showed a small, significant relationship with life satisfaction but not quality of life, while gender was unrelated to both. Being married or in a de facto relationship was positively related to both well-being measures, while only quality of life was significantly related to being in paid work. The positive intercorrelations between the SES, health and well-being indicators were all significant except for the correlation between parental SES and life satisfaction. The adult SES and health indicators generally had larger correlations with both well-being measures than the childhood indicators, supporting Hypothesis 2.

**Table 1:** Means, Standard Deviations (SD), and intercorrelations among study variables

Variable	Mean (SD)		1	2	3	4	5	6	7	8	9	10	11	12	13
1. Age	61.38 (1.27)														
2. Gender	54 % male		0.02												
3. Marital status	82 % partnered		−0.01	−0.10**											
4. Work status	54 % working		−0.16***	−0.11***	−0.03										
5. Parental SES	46.94	(22.00)	−0.02	0.07*	−0.03	0.05									
6. Books at home	2.73	(1.22)	−0.02	0.08**	0.01	0.05	0.35***								
7. School at age 16	0.65	(0.48)	−0.12***	−0.04	0.01	0.05	0.25***	0.29***							
8. Highest education	1.05	(0.73)	−0.03	−0.04	0.02	0.10***	0.21***	0.22***	0.36***						
9. Most significant job	58.11	(21.30)	−0.06*	0.00	0.05	0.08**	0.24***	0.21***	0.37***	0.53***					
10. Household income	6.36	(1.76)	−0.07*	−0.11***	0.21***	0.26***	0.19***	0.17***	0.28***	0.35***	0.40***				
11. Childhood health	1.83	(0.96)	0.04	0.02	−0.01	−0.06*	−0.05	−0.05	−0.12***	−0.06*	−0.12***	−0.16***			
12. Adult health	2.43	(0.94)	0.04	−0.06*	−0.08**	−0.16***	−0.13***	−0.09**	−0.17***	−0.20***	−0.22***	−0.28***	0.32***		
13. Quality of life	44.51	(8.05)	0.05	0.02	0.13***	0.10****	0.09***	0.13***	0.13***	0.21***	0.23***	0.29***	−0.21***	−0.56***	
14. Life satisfaction	27.16	(5.85)	0.08**	−0.04	0.25***	0.04	0.04	0.07**	0.11***	0.13***	0.16***	0.25***	−0.16***	−0.41***	0.75***

Gender coded 0 = male, 1 = female; Marital status coded 0 = not married/de facto, 1 = married/de facto; Work status coded 0 = not in paid work, 1 = in paid work

*SES* Socioeconomic status

*p < .05, **p < .01, ***p < .001 (two-tailed)

To test Hypotheses 3 and 4, we first examined a full mediation model (1) that omitted the pathways from childhood SES to adult health and from childhood health to adult SES. As seen in Table 2, the fit statistics for full mediation model 1 indicated relatively good model fit. Next, we tested a partial mediation model that added a direct link from each childhood SES factor and childhood health to both quality of life and life satisfaction. The partial mediation model did not significantly improve fit, as indicated by the non-significant chi-square change statistic, ∆*χ*^2^ (8) = 11.65, *p* > .05, higher RMSEA, and AIC values shown in Table 2. The only childhood factor that had a significant direct effect was from books at home to higher quality of life (*β* = .06, *p* = .015). A second full mediation model (2a) added the direct pathway from books at home to quality of life. This significantly improved the fit of the first model, ∆*χ*^2^ (1) = 3.97, *p* = .046, and this direct effect was retained in subsequent models. These results suggest that consistent with Hypotheses 3 and 4, adult SES mediates the effects of childhood SES on later well-being, and adult health mediates the effects of childhood health on well-being.

**Table 2:** Fit statistics of mediated models of Socioeconomic Status (SES), health and well-being

Model	*χ* ^2^	*df*	*p*	RMSEA	CFI	SRMR	AIC
1. Full mediation 1	70.57	14	<0.001	0.06	0.98	0.03	36359.43
2. Partial mediation	58.92	6	<0.001	0.08	0.98	0.03	36363.78
3. Full mediation 2a	66.60^a^	13	<0.001	0.06	0.98	0.03	36357.46
4. Full mediation 2b	30.54^b^	10	0.001	0.04	0.99	0.01	36327.41
5. Full mediation 2c	7.69^b^	7	0.361	0.01	1.00	0.01	36310.55

Full mediation 1: Drop direct effects from childhood SES (parental SES, books at home, school at age 16) and childhood health to well-being (quality of life or life satisfaction). Partial mediation: Add direct effects from childhood SES and health to well-being. Full mediation 2a: Add direct effect from books at home to quality of life. Full mediation 2b: Add direct effects from childhood SES to adult health. Full mediation 2c: Add direct effects from childhood health to adult SES

*RMSEA* root mean square error of approximation, *CFI* comparative fit index, *SRMR* standardized root square residual, *AIC* Akaike information criterion

^a^Difference in chi-square from the preceding full mediation model is significant at *p* < .05 (one-tailed)

^b^Difference in chi-square from the preceding full mediation model is significant at *p* < .001 (one-tailed)

To examine whether adult health mediates the effects of childhood SES, the full mediation model 2b added three direct paths linking each childhood SES factor to adult health. Model 2b fit the data significantly better than model 2a, ∆*χ*^2^ (3) = 36.06, *p* < .001, supporting the pathway from childhood SES to adult health proposed in Hypothesis 4. Finally, to test whether adult SES mediates the effect of childhood health (Hypothesis 3), three direct paths linking childhood health to each adult SES factor were added in model 2c, which provided the best fit for the data, ∆*χ*^2^ (3) = 22.85, *p* < .001. The variance explained by this model was as follows: highest education, *R*^2^ = 16 %; most significant job, *R*^2^ = 18 %; household income, *R*^2^ = 22 %; adult health, *R*^2^ = 16 %; life satisfaction, *R*^2^ = 24 %; and quality of life, *R*^2^ = 36 %.

Standardized parameter estimates for the final full mediation model 2c are shown in Fig. 1. Although not shown in Fig. 1, age, gender, marital status, and work status were entered as control variables on which each endogenous variable was regressed. In this model, age was positively related to both quality of life and life satisfaction, while gender was related to adult health and household income, with women reporting better health and lower household incomes. Those who were married or in a de facto relationship tended to have higher adult SES levels, indicated by their most significant job and household income, better health, quality of life, and life satisfaction. Those in paid work tended to have higher educational levels, household incomes, and better health.

As Fig. 1 shows, all paths from childhood to adult SES and health were significant except for books at home to adult health and childhood health to highest education. Two adult SES factors, most significant job and household income, were significantly related to quality of life, but only household income was related to life satisfaction, partly supporting Hypothesis 3. The significance of standardized indirect effects was tested using 95 % confidence intervals derived from bias-corrected bootstrap estimates in Mplus. This approach is recommended over more common approaches such as the Sobel test or causal steps approach as it has higher power while maintaining reasonable control over the Type I error rate (Cheung and Lau 2008; MacKinnon et al. 2004; Preacher and Hayes 2008). The indirect effects are considered significant when the confidence interval (CI) does not include zero.

Bootstrap analyses revealed significant indirect effects from parental SES to both quality of life and life satisfaction through household income, 95 % CIs [0.002, 0.019], and [0.002, 0.020], respectively, and through adult health, 95 % CIs [0.013, 0.069], and [0.009, 0.049], respectively. Indirect effects from being at school at age 16 through household income and adult health were significant for both quality of life, 95 % CIs [0.007, 0.033], and [0.030, 0.089], respectively, and life satisfaction, 95 % CIs [0.005, 0.034], and [0.021, 0.064], respectively. Childhood health also had significant indirect effects to quality of life and life satisfaction through household income, 95 % CIs [−0.019, −0.003], and [−0.020, −0.002], respectively, and adult health, 95 % CIs [−0.184, −0.120], and [−0.136, −0.082], respectively. There were no significant indirect effects of books at home on quality of life or life satisfaction. The estimates as well as CIs for all the indirect effects are provided as an online supplement in Table A1.

## Discussion

This study examined pathways through which earlier childhood and adult socioeconomic and health circumstances impact on the subjective well-being of Australia’s large baby boom cohort now entering later life. Overall, the results supported the view that childhood SES and health matter, albeit indirectly, with more proximal adult SES and health factors significantly mediating the effects of childhood SES and health on well-being in later life. The findings help explain some of the mixed results in the literature on the effects of childhood SES on well-being among older adults and support a pathway model of life course influences on subjective well-being that includes both SES and health factors. Extending prior research that focused on more objective health measures and consistent with emerging European research on well-being (Deindl 2013; Wahrendorf and Blane 2014), we found support for similar causal mechanisms through which early socioeconomic and health exposures impact on health, education and social class attainment in midlife, and subsequent well-being on entry to later life.

Following recommendations by Niedzwiedz et al. (2012), the current study tests hypotheses derived from three prominent life course models, the accumulation model (Hypothesis 1), the critical period model (Hypothesis 2), and a pathway model (Hypotheses 3 and 4). We found support for all three models, consistent with the emergent view that these models are not necessarily incompatible, and can be used together to shed light on different and complementary aspects of the research question.

Consistent with Hypothesis 1 and prior research supporting the accumulation model (Wahrendorf and Blane 2014), the number of negative SES and health exposures across the life course was positively related to poorer well-being outcomes among older adults. Supporting Hypothesis 2 and prior research (Deindl 2013; Wahrendorf and Blane 2014), bivariate analyses showed that more proximal adult factors were more strongly related to later life well-being than more distal childhood factors. The multivariate analyses supported and qualified this finding by showing that, while adult SES and health have significant direct effects on well-being, childhood factors including parental SES, remaining in school at age 16, and childhood health have significant indirect effects. Supporting Hypotheses 3 and 4, the effects of childhood exposures, specifically, parental SES, school at age 16, and childhood health, on later well-being were fully mediated by adult exposures, specifically, household income and adult health. The lack of a statistically significant path from highest education to well-being warrants some discussion. As Table 1 shows, highest education had slightly smaller correlations with both well-being measures than did household income and most significant job, possibly because it is likely to be the most distal adult SES indicator. In addition, it was moderately correlated with both most significant job (*r* = .53) and household income (*r* = .35), so it is possible that the relatively smaller effects of highest education on well-being are further reduced when the most significant job and income variables are included in the same model.

Extending our knowledge of the social determinants of health to well-being, we found that adult health mediates the effects of childhood SES on well-being. This finding is consistent with our longitudinal research with an earlier cohort of older people, which found that continuing action to enhance healthy lifestyles can contribute to maintaining health and well-being throughout later life (Kendig et al. 2014a). We also found a significant pathway from childhood health to adult SES to well-being. While past research has indicated that both adult SES and health significantly mediated the effects of childhood factors (Haas et al. 2012), this study sheds additional light on the interactive pathways between SES and health over the life course.

The final model explained more variance in quality of life (36 %) than life satisfaction (24 %), childhood exposures tended to correlate more strongly with quality of life than life satisfaction, and books at home even had a direct effect in the multivariate analysis, suggesting that one’s early socioeconomic exposures may have more differential impacts on eudemonic than hedonic well-being later in life. One possible explanation for this effect might be fundamental differences in the determinants of these well-being outcomes. Eudemonic well-being reflects higher-level, value-laden, existential evaluations of self-realization, control and autonomy (Jivraj et al. 2014; Ryff et al. 2003) that are likely to depend more on experiences and achievements over time. In contrast, hedonic well-being may behave more like inherent personality traits that are less reflective of external life circumstances and achievements (Cummins 2010; Plagnol 2010). An important implication of this finding is that future research should consider the domain of well-being being assessed and how this might affect the findings and recommendations. This research also supports Niedzwiedz et al.’s (2012) suggestion that the mixed findings in the literature may be attributable in part to the use of different well-being measures.

A few limitations of this study should be discussed. First, data on childhood and adult exposures across the life course were collected retrospectively, raising concerns about recall bias. For example, it is possible that one’s current level of subjective well-being influences recollections and evaluations of past events (O’Brien et al. 2012). However, prospective data collection is not possible once the cohort of interest is already older unless other sources of life history data are readily available (Kendig et al. 2014b). Thus, collecting retrospective life course data, reinforced by event history calendars to aid accurate recall (Belli et al. 2007; Berney and Blane 1997), enables us to analyze processes and pathways relevant to this early baby boomer cohort that may otherwise have remained unexplored. In addition, past research (e.g., Haas 2007; Hardt et al. 2006; Havari and Mazzonna 2011) has provided evidence of the reliability and validity of retrospective data.

It is also possible that other indicators of childhood and adult exposures not collected in this study, such as parental education and family income in childhood, may have stronger predictive validity than parental occupation for well-being outcomes later in life. Future research could examine the role of pathways other than SES and health, by including other potentially relevant adverse childhood exposures such as abuse, family conflict and disruption (Blane et al. 2012; Brandt et al. 2012). Pathways may also include advantageous exposures such as childhood happiness (Bellis et al. 2013), parental care (Huppert et al. 2010), and cultural capital (De Graaf et al. 2000; Jæger 2009), which may partly explain the direct effect books at home had on later quality of life (Cavapozzi et al. 2011). The effect of books on later quality of life is particularly interesting and is consistent with prior research showing that this marker of cultural capital confers additional advantages beyond parental education, occupation and social class, perhaps because it reflects, alongside other advantages, a scholarly culture including the promotion of skills and competencies that are directly useful for learning and education, and a preference for and enjoyment of scholarly pursuits (Evans et al. 2010). Cognitive resources and personality factors such as openness to experience, creativity, resilience and locus of control may also be linked to alternative pathways to well-being later in life (Pearlin 2010). A more complete model including these and other potentially relevant factors or covariates may alter the substantive findings on the relative effects of childhood and adult SES and health exposures on well-being later in life.

Our use of two established well-being measures that have been used in large-scale longitudinal ageing studies such as ELSA and SHARE, enables comparisons with other research and may be regarded as a strength of the study. However, future research may benefit from including well-being on other life domains such as work, family, friends, and leisure (Huppert and So 2013; Plagnol 2010; Schafer et al. 2013).

More fundamental issues arise in understanding the validity and meaning of retrospective life history methods. Of particular importance for this paper is the retrospective self-assessed health indicator. Given the possibility that perceptions of health are influenced by respondents’ socioeconomic position (perhaps as a result of current health), the empirical relationship between the two could reflect some confounding. Further temporal concerns arise as to the age- and class-related interpretations and expectations for states of health and well-being for the future as well as the present and past. These difficulties extend well beyond matters of selective recall or sample attrition. They are accentuated when self-reports are sought over long periods of time during which there would be major life span and historically grounded changes in people’s expectations for health, the saliency of different dimensions of health for their lives, and variation in the work, family, and personal context of their lives. Future research could explore key factors in these relationships and increase understanding of complex contextual influences on health and well-being.

Finally, the generalizability of this research should be considered given our use of an Australian sample aged 60 to 64 years, predominantly from Anglo or Western cultural backgrounds, and with relatively higher educational qualifications and self-rated health than the Australian population (Kendig et al. 2014b). It is possible that the effects of life course exposures have been attenuated in the present study due to restriction of the range and relatively fewer negative exposures, so future research should include a more representative sample of less privileged older populations.

In conclusion, this study applied a relatively novel approach to examine the pathways through which childhood factors can impact later life well-being. It showed that both childhood and adult SES and health are important for the subjective well-being of Australian baby boomers. In addition to supporting the accumulation model in which SES and health exposures over the life course will have cumulative effects on well-being in later life, our findings also highlight the importance of the timing of these exposures and how childhood effects on both the quality of life and life satisfaction in later life are largely mediated by adult exposures. While adult exposures had stronger and direct effects on subjective well-being, the indirect effects of childhood factors underscore the value in implementing actions that reduce negative exposures or strengthen resilience across the life course. Broadly, the findings reinforce the fundamental value of policies addressing the challenges and opportunities of societal ageing through building personal capacities early in life and reinforcing them throughout middle and later life.

## Electronic supplementary material

ESM 1 (DOCX 31 kb)
