# Supplementary material for: Pathways to Well-Being in Later Life: Socioeconomic and Health Determinants Across the Life Course of Australian Baby Boomers
Source: J Popul Ageing. 2015 Aug 19;9:49–67. doi: 10.1007/s12062-015-9132-0 (PMC4785210; doi:10.1007/s12062-015-9132-0)
Supplement: Supplementary file 1 — (DOCX 31 kb) [file 12062_2015_9132_MOESM1_ESM.docx]

**Appendix: Supplementary Material**

Table A1

*Estimates and Confidence Intervals (CI) of Standardized Specific Indirect Effects from Childhood Indicators to Well-being in Later Life via Adult Indicators*

|  | Quality of Life | | | Life Satisfaction | | |
| --- | --- | --- | --- | --- | --- | --- |
|  | Indirect | 95% CI | | Indirect | 95% CI | |
| Specific Pathway | effect | Lower | Upper | effect | Lower | Upper |
| Parental SES |  |  |  |  |  |  |
| Highest education | 0.004 | -0.003 | 0.010 | 0.001 | -0.005 | 0.008 |
| Most significant job | 0.008 | -0.001 | 0.017 | 0.004 | -0.005 | 0.013 |
| Household income | 0.011 | 0.002 | 0.019 | 0.011 | 0.002 | 0.020 |
| Adult health | 0.041 | 0.013 | 0.069 | 0.029 | 0.009 | 0.049 |
| Books at home |  |  |  |  |  |  |
| Highest education | 0.003 | -0.003 | 0.009 | 0.001 | -0.005 | 0.007 |
| Most significant job | 0.003 | -0.002 | 0.009 | 0.002 | -0.003 | 0.006 |
| Household income | 0.006 | 0.000 | 0.012 | 0.006 | -0.001 | 0.012 |
| Adult health | 0.000 | -0.029 | 0.028 | 0.000 | -0.021 | 0.020 |
| School at age 16 |  |  |  |  |  |  |
| Highest education | 0.011 | -0.007 | 0.028 | 0.004 | -0.015 | 0.023 |
| Most significant job | 0.017 | -0.002 | 0.036 | 0.009 | -0.011 | 0.028 |
| Household income | 0.020 | 0.007 | 0.033 | 0.020 | 0.005 | 0.034 |
| Adult health | 0.059 | 0.030 | 0.089 | 0.042 | 0.021 | 0.064 |
| Childhood health |  |  |  |  |  |  |
| Highest education | 0.000 | -0.003 | 0.002 | 0.000 | -0.002 | 0.002 |
| Most significant job | -0.004 | -0.009 | 0.002 | -0.002 | -0.006 | 0.003 |
| Household income | -0.011 | -0.019 | -0.003 | -0.011 | -0.020 | -0.002 |
| Adult health | -0.152 | -0.184 | -0.120 | -0.106 | -0.136 | -0.082 |
